# Social media exposure and its association with acceptance of cosmetic surgery among adult females in Jazan

**DOI:** 10.3389/fpubh.2026.1871180

**Published:** 2026-07-31

**Authors:** Abdullah A. Alharbi, Mohammed A. Muaddi, Anas E. Ahmed, Ahmad Assiri, Amr Youssef Arkoubi, Taif I. Alameer, Shahad M. Alharbi, Afnan H. Abuzawah, Renad A. Madkhali, Sawsan A. Refaei, Raghd A. Babaeer, Amani A. Gharwi, Amjad H. Muthaffar, Ahmad Y. Alqassim

**Affiliations:** 1 Department of Family and Community Medicine, College of Medicine, Jazan University, Jazan, Saudi Arabia; 2 Dermatology Unit, Department of Internal Medicine, College of Medicine, Jazan University, Jazan, Saudi Arabia; 3 Department of Anesthesia and Surgery, College of Medicine, Imam Mohammad Ibn Saud Islamic University (IMSIU), Riyadh, Saudi Arabia; 4 College of Medicine, Jazan University, Jazan, Saudi Arabia

**Keywords:** beauty standards, cosmetic surgery acceptance, Jazan, perception, Saudi Arabian women, social media influence

## Abstract

**Background:**

Social media exposure may be associated with women's attitudes toward cosmetic surgery, yet data from Saudi Arabia's Jazan region are lacking.

**Objectives:**

To evaluate acceptance of cosmetic surgery and its association with social media exposure among adult women in Jazan.

**Methods:**

This cross-sectional study recruited 1,206 women aged ≥18 years via WhatsApp and Snapchat between 1 and 30 January 2025. An Arabic Google Forms questionnaire included the validated Acceptance of Cosmetic Surgery Scale (ACSS), sociodemographic items, and social media behaviors. Multivariable linear regression identified factors associated with ACSS score.

**Results:**

Mean ACSS score was 58.90 (SD 25.07). Higher acceptance was observed among older participants (≥45 years: β = 11.62, 95% CI 5.32–17.92, *p* < 0.001), higher income (>10,000 SAR: β = 4.39, *p* = 0.038), and each additional hour of daily social media use (β = 0.38, *p* = 0.035). Following beauty experts showed the strongest effect (β = 11.85, 95% CI 8.43–15.27, *p* < 0.001), followed by using photo-editing apps (β = 5.33, *p* < 0.001), comparing appearance with influencers (β = 3.81, *p* = 0.017), and posting selfies (β = 3.70, *p* = 0.012).

**Conclusions:**

Social media engagement and demographic factors were associated with cosmetic surgery acceptance among women in Jazan. These associations may have implications for media-literacy interventions, transparent content regulation, and ethical pre-procedure counseling. However, as recruitment occurred via social media apps, results may not generalize to all women in the region.

## 1 Introduction

Social media enables people to share content and experiences with a wide audience almost instantly (1), and its use has grown rapidly, with the number of users nearly doubling from 2.2 billion in 2015 to 4.5 billion in 2022 (2). In Saudi Arabia, social media is a common part of daily life, especially among young adults; in 2023, over 90% of those aged 15–34 reported social networking as their most frequent online activity (3). Social media content significantly relates to people's attitudes toward cosmetic surgery, defined as any surgical procedure primarily aimed at enhancing physical appearance, which ranks among the most frequently performed medical procedures (4). Social media has become increasingly prevalent in contemporary society, influencing individuals' daily decisions and perceptions. Platforms such as Facebook, Instagram, Twitter, Snapchat, and more recently TikTok are widely used worldwide (5).

Social media may shape these attitudes through direct promotion by influencers, digital filters that reinforce unrealistic beauty standards, and the sharing of cosmetic experiences online (6–8). Globally, cosmetic procedures rose by 3.4% in 2023, reaching approximately 34.9 million surgical and non-surgical procedures (9). In Saudi Arabia, a cross-sectional study found that 48.5% of respondents were influenced by advertisements to consider cosmetic treatments (6), and the desire for cosmetic surgery rises with social media use, with prevalence estimates ranging from 21 to 55% (1, 6, 10). Commonly reported drivers include influencer content, before-and-after images, and the desire to enhance one's own selfies (7, 11, 12). Additionally, a study in Iran indicated that there is a significant relationship between economic status and cosmetic surgery, the incidence was 5.6 times higher among those of average status and 3.14 times higher among those of wealthy status compared with poor economic status (9, 13). In another Saudi study, the participants reported that 43.5% had a normal BMI, 37.8% were obese, and 18.7% were overweight. Among them, 41% had undergone plastic surgery (14).

However, most Saudi research has focused on the central and eastern regions, leaving the southwestern Jazan region; where sociocultural norms may differ—largely unstudied. Clarifying this relationship is of public health relevance, as it can inform media-literacy education, ethical pre-procedure counseling, and regulation of misleading cosmetic content, thereby supporting informed decision-making among women.

Therefore, this cross-sectional study aimed to assess acceptance of cosmetic surgery and examine its association with social media exposure among women aged 18 years and older in Jazan. In this study, the primary outcome—assessed using the Acceptance of Cosmetic Surgery Scale (ACSS); refers specifically to the acceptance of cosmetic surgery (i.e., surgical procedures intended to enhance appearance). The broader term “cosmetic procedures” is used only when describing previous studies that examined both surgical and non-surgical (minimally invasive) procedures. By investigating this relationship, our study seeks to highlight the potential association of social media with women's healthcare-related attitudes and decisions. Understanding how social media relates to women's perceptions of cosmetic procedures can help healthcare professionals and policymakers develop targeted educational interventions that promote informed decision-making. Furthermore, it may support the creation of guidelines or regulations aimed at reducing misinformation about cosmetic enhancements.

## 2 Materials and Methods

### 2.1 Study procedure

This cross-sectional study involved 1,206 participants from Jazan, a southwestern region of Saudi Arabia. Data collection was conducted from January 1 to 30, 2025. The survey was distributed electronically via social media platforms, primarily WhatsApp and Snapchat, in Arabic language. This distribution strategy was selected because over 95% of the target age group regularly uses social media in Saudi Arabia (15). The sample included female Jazan residents aged 18 and older. Prior to starting the online questionnaire, participants read the study objectives and provided electronic informed consent before accessing the survey.

### 2.2 Sample size, sampling, and data collection

The sample size was calculated using the standard cross-sectional formula: *n* = $Z_{1-\alpha }^{2}$ × *P* (1 – *P*)/*d*^2^, with *Z* = 1.96 (95% confidence level), *P* = 0.5 (conservative estimate without prior data on cosmetic surgery acceptance), and *d* = 0.05 (margin of error), yielding 384 participants. Adding 10% for non-response yielded 423 participants.

Convenience sampling through social media platforms, primarily WhatsApp, targeted adult women (aged ≥18 years) in Jazan region. This approach resulted in 1,206 completed responses, nearly tripling our target sample size. While non-probability sampling has limitations, the high response rate enhanced result reliability and statistical power (16), providing sufficient data to examine the relationship between social media exposure and cosmetic surgery acceptance.

Data was collected using a self-administered online questionnaire via Google Forms, selected for its user-friendly interface and accessibility. This platform allowed convenient questionnaire completion while preserving participant anonymity by collecting no identifying information. The survey link was distributed through WhatsApp groups and other social media platforms to reach a diverse audience. The study gathered 1,206 complete responses, exceeding the calculated sample size requirement.

### 2.3 Measurements

All data was gathered through a self-administered Arabic online questionnaire covering socioeconomic and demographic characteristics, social media-related behaviors, and attitudes toward cosmetic surgery. The measurement comprised 3 parts: The first part was the Acceptance of Cosmetic Surgeries Scale (ACSS), which served as the dependent variable and outcome measure. This continuous variable measured cosmetic surgery acceptance using the Arabic version of the ACSS, a widely validated screening tool. This scale was validated in a Saudi general population with a Cronbach's α = 0.912, indicating high reliability (17). In the current study, the Arabic version of the ACSS demonstrated a high level of internal consistency (Cronbach's α = 0.95), supporting the reliability of the instrument among adult females in Jazan.

The ACSS consists of 15 items measured on a 7-point Likert scale (1 = strongly disagree to 7 = strongly agree), with total scores ranging from 15 to 105. Higher scores reflect greater acceptance of cosmetic surgery. The scale divides into three subscales: Interpersonal (evaluating self-oriented benefits), Social (examining social motivations), and Consideration (assessing whether respondents would consider undergoing cosmetic surgery) (18, 19).

The remaining parts measured independent variables. The socioeconomic and demographic section included questions about age, nationality, BMI, marital status, income range, employment status, and educational status. BMI was self-reported by participants and calculated as weight (kg)/height (m)^2^. Based on the World Health Organization (WHO) classification (20), BMI categories were: underweight < 18.5 kg/m^2^, normal 18.5–24.9 kg/m^2^, overweight 25–29.9 kg/m^2^, and obese ≥30 kg/m^2^. The social media section comprised 12 questions assessing social media's influence on participants' self-perception and behaviors, including time spent on platforms, selfie posting frequency, filter/editing use, comparison behaviors, following patterns (plastic surgeons/fashionistas), and social media's effects on beauty perceptions, fitness interests, and plastic surgery advertisements. This section also explored how followed accounts influenced participants' self-perception (1, 21–25).

### 2.4 Ethical considerations

This study was approved by the Jazan University Standing Committee for Scientific Research (Reference No.: REC-46/06/1280, 24 December 2024). Prior to starting the online questionnaire, each participant provided explicit electronic informed consent by selecting an “I agree to participate” option after reading the study objectives. Participation was voluntary, and participants could withdraw at any time before submitting their responses. All data were collected anonymously and used solely for scientific research in accordance with ethical standards.

### 2.5 Statistical analysis

The unit of analysis was the individual adult female resident of the Jazan region, and all data were obtained through the validated Arabic online questionnaire. Data were analyzed in three steps. First, the sample was described using frequencies and percentages for categorical variables and medians with interquartile ranges for continuous variables. Second, median ACSS scores were compared across sociodemographic and social media–related subgroups using the Mann–Whitney *U*-test and the Kruskal–Wallis test. Third, a multivariable linear regression model was fitted to identify factors independently associated with the ACSS score, reporting coefficients (β) with 95% confidence intervals. Categorical predictors were dummy-coded (reference = lowest or most frequent category), and regression assumptions were assessed. Given heteroscedasticity on the Breusch–Pagan test, heteroscedasticity-robust (Huber–White) standard errors were applied; multicollinearity was evaluated using variance inflation factors (VIFs), and model fit using *R*^2^ and adjusted *R*^2^. Analyses were performed in Stata version 16; all tests were two-sided, with *p* < 0.05 considered statistically significant.

## 3 Results

The 1,206 participants were predominantly young (75.1% aged ≤ 34 years), single, and university-educated, with most reporting a monthly income below SAR 5,000 (Table 1). Social media use was high (mean 6.47 h/day, SD 3.82), and Snapchat and TikTok were the dominant platforms. About one-third had posted selfies in the past month, and roughly one in five followed beauty experts. The overall mean ACSS score was 58.90 (SD 25.07).

**Table 1:** Sociodemographic characteristics and social media usage patterns among female participants in the Jazan region, Saudi Arabia (*N* = 1,206).

Characteristic	*n* (%)
Age, years	
18–23	663 (55.0)
24–34	243 (20.2)
35–44	160 (13.3)
≥45	140 (11.6)
Marital status	
Single	739 (61.3)
Married	418 (34.7)
Divorced	41 (3.4)
Widowed	8 (0.7)
Education	
Secondary or less	224 (18.6)
Bachelor's	937 (77.7)
Postgraduate	45 (3.7)
Monthly income, SAR	
< 5,000	843 (69.9)
5,000–10,000	135 (11.2)
>10,000	228 (18.9)
BMI category	
Underweight	201 (16.7)
Normal	576 (47.8)
Overweight	259 (21.5)
Obese	170 (14.1)
Most-used platform	
Facebook	28 (2.3)
X (Twitter)	95 (7.9)
Instagram	197 (16.3)
Snapchat	425 (35.2)
TikTok	461 (38.2)
Posted selfies in past month	
No	767 (63.6)
Yes	439 (36.4)
Follows beauty experts	
No	973 (80.7)
Yes	233 (19.3)
Wants influencer look	
No	925 (76.7)
Yes	281 (23.3)
Reason for following influencers	
Does not follow	644 (53.4)
Beauty, fitness, cosmetic content	188 (15.6)
Daily routine, lifestyle	374 (31.0)
Wants to look like influencers^‡^	
No	867 (71.9)
Yes	339 (28.1)
Photo editing increases picture sharing	
No	835 (69.2)
Yes	371 (30.8)
**Daily social media use, h, mean (SD)**	6.47 (3.82)
**ACSS score, mean (SD)**	58.90 (25.07)

Data are *n* (%) unless otherwise indicated. Percentages may not total 100 due to rounding. BMI, body mass index; ACSS, acceptance of cosmetic surgery scale; SAR, Saudi Riyal.

^‡^See flag 2 below.

In unadjusted analyses, all sociodemographic and social media variables were significantly associated with the ACSS score (all *p* ≤ 0.02; Table 2). Acceptance rose steadily with age and income and was higher among divorced and postgraduate women. Among social media factors, the widest gaps were seen for following beauty experts, wanting to resemble influencers, following influencers for beauty or fitness content, and using photo-editing apps, each showing markedly higher median scores in the exposed group; Snapchat users reported the highest platform-specific scores.

**Table 2:** Median of acceptance of cosmetic surgery scale (ACSS) scores by female characteristics in Jazan region, Saudi Arabia (*N* = 1,206).

Characteristic	Median (IQR)	*p*-value^†^
**Age, years**		**<0.001**
18–23	55 (35–74)	
24–34	64 (42–83)	
35–44	69 (48.5–86)	
≥45	71 (46–85)	
**Marital status**		**<0.001**
Single	56 (35–75)	
Married	66 (42–82)	
Divorced	75 (60–91)	
Widowed	60.5 (49–71)	
**Education**		**0.020**
Secondary or less	57.5 (33.5–75)	
Bachelor's	60 (38–78)	
Postgraduate	68 (49–86)	
**Monthly income, SAR**		**<0.001**
<5,000	57 (34–75)	
5,000–10,000	65 (45–82)	
>10,000	69.5 (49.5–84)	
**BMI category**		**0.005**
Underweight	55 (39–73)	
Normal	60 (35–77)	
Overweight	64 (43–82)	
Obese	62 (38–82)	
**Most-used platform**		**<0.001**
Facebook	50 (31–74)	
X (Twitter)	51 (32–75)	
Instagram	51 (30–70)	
Snapchat	65 (43–84)	
TikTok	60 (39–75)	
**Posted selfies in past month**		**<0.001**
No	59 (35–75)	
Yes	63 (46–82)	
**Follows beauty experts**		**<0.001**
No	56 (33–75)	
Yes	75 (60–89)	
**Wants to look like influencers**		**<0.001**
No	58 (34–75)	
Yes	73 (53–90)	
**Reason for following influencers**		**<0.001**
Does not follow	55 (32–75)	
Beauty, fitness, cosmetic content	75 (60–89)	
Daily routine, lifestyle	60 (41–77)	
**Compares appearance with influencers**		**<0.001**
No	59 (33–75)	
Yes	68 (48–88)	
**Photo editing increases picture sharing**		**<0.001**
No	57 (33–75)	
Yes	73 (49–86)	

IQR, interquartile range.

^†^From Mann–Whitney U (two groups) or Kruskal–Wallis (>two groups) tests; the value shown applies to the whole variable. BMI, body mass index; SAR, Saudi Riyal. Bold values indicate statistically significant results (*p* <0.05).

Table 3 presents the multivariable linear regression. After adjustment, ACSS scores rose with age relative to the youngest group (18–23 years), increasing by 4.56 points at 24–34 years (*p* = 0.030), 9.97 at 35–44 years (*p* = 0.001), and 11.62 at ≥45 years (*p* < 0.001). Higher income was also independently associated with acceptance: women earning >10,000 SAR scored 4.39 points higher than those earning < 5,000 SAR (*p* = 0.038). Several social media factors remained independently associated with ACSS score. Each additional hour of daily use was associated with a 0.38-point increase (*p* = 0.035). Relative to Instagram users, Snapchat (β = 4.55, *p* = 0.026) and TikTok (β = 4.34, *p* = 0.024) users scored higher. The strongest associations were following beauty experts (β = 11.85, *p* < 0.001), wanting to look like influencers (β = 7.97, *p* < 0.001), and following influencers for beauty, fitness, or cosmetic content (β = 7.99, *p* < 0.001), followed by photo-editing app use (β = 5.33, *p* < 0.001), appearance comparison with influencers (β = 3.81, *p* = 0.017), and selfie posting (β = 3.70, *p* = 0.012).

**Table 3:** Multivariable linear regression of factors associated with the acceptance of cosmetic surgery scale (ACSS) score (*N* = 1,206).

Predictor	β	95% CI	*p*-value^†^
Age, years (ref: 18–23)			
24–34	4.56	0.44 to 8.68	0.030
35–44	9.97	4.30 to 15.63	0.001
≥45	11.62	5.32 to 17.92	< 0.001
Marital status (ref: single)			
Married	2.29	−1.43 to 6.01	0.227
Divorced	−0.77	−8.43 to 6.89	0.844
Widowed	5.53	−10.64 to 21.71	0.502
Monthly income, SAR (ref:<5,000)			
5,000–10,000	1.85	−2.89 to 6.59	0.444
>10,000	4.39	0.25 to 8.54	0.038
BMI category (ref: normal)			
Underweight	1.97	−1.73 to 5.67	0.297
Overweight	0.62	−2.84 to 4.08	0.725
Obese	−2.18	−6.39 to 2.04	0.312
Daily social media use (per h)	0.38	0.03 to 0.74	0.035
Most-used platform (ref: Instagram)			
Facebook	−7.47	−16.93 to 1.99	0.122
X (Twitter)	0.74	−4.78 to 6.26	0.792
Snapchat	4.55	0.54 to 8.55	0.026
TikTok	4.34	0.58 to 8.09	0.024
Posted selfies in past month (ref: no)			
Yes	3.70	0.82 to 6.58	0.012
Follows beauty experts (ref: no)			
Yes	11.85	8.43 to 15.27	< 0.001
Wants to look like influencers (ref: no)			
Yes	7.97	4.58 to 11.36	< 0.001
Reason for following influencers (ref: does not follow)			
Beauty, fitness, cosmetic content	7.99	3.92 to 12.05	< 0.001
Daily routine, lifestyle	0.99	−2.07 to 4.04	0.527
Compares appearance with influencers (ref: no)			
Yes	3.81	0.69 to 6.93	0.017
Photo editing increases picture sharing (ref: no)			
Yes	5.33	2.35 to 8.31	< 0.001

β, unstandardized regression coefficient; CI, confidence interval; BMI, body mass index; SAR, Saudi Riyal. †Two-sided; *p* < 0.05 considered significant. The model was significant and explained 22.5% of the variance in ACSS scores (*R*^2^ = 0.225, adjusted *R*^2^ = 0.210; F (23, 1182) = 21.52, *p* < 0.001). Residuals were approximately normal with no influential observations and no problematic multicollinearity (mean VIF = 1.51); heteroscedasticity-robust (Huber–White) standard errors were applied, and the direction and significance of all associations were unchanged.

## 4 Discussion

The study aimed to assess the acceptance of cosmetic surgery and examine its association with social media use among women aged 18 years and older in the Jazan region. We found a relatively high acceptance level (mean: 58.90), slightly higher than a previous national study reporting 55.4% in Eastern regions (17). This difference likely reflects regional socio-cultural variations. Age, income, and social media behaviors including selfie posting, following beauty influencers, and using photo-editing apps significantly correlated with increased cosmetic surgery acceptance.

Regarding age, our study found that acceptance was higher among older women, with the highest levels observed among those aged ≥45 years compared with those aged 18–24. These findings align with previous research in Saudi Arabia. A study in the Eastern region (2021) reported significantly higher acceptance among participants over 45 compared to younger individuals (mean scores: 66.1 vs. 51.9, *P* = 0.001) (17). Similarly, research in Riyadh and Jeddah (2016) found that older individuals sought cosmetic procedures to avoid age discrimination and maintain youthfulness (26). Recent studies in Makkah and Medina (2023) confirmed higher ACSS scores among individuals over 30 (27). However, our results contradict findings from Mosul, Iraq (2023), which reported younger individuals (18–30) were more likely to consider cosmetic procedures (19). Studies in Belgrade, Serbia (2019), China and the Netherlands (2021) found no strong correlation between age and acceptance (28, 29). These contradictions may reflect cultural variations in beauty standards, with Western societies having different expectations than regions like Saudi Arabia, where societal pressures might increase with age.

Our study revealed a significant correlation between higher income and increased acceptance of cosmetic surgery, with women earning over 10,000 SAR scoring 4.39 points higher on the ACSS scale. These findings align with research conducted in Riyadh and Jeddah (2016), which demonstrated that higher economic status correlates with greater access to cosmetic procedures, with wealthier individuals more likely to undergo procedures due to their financial means (26). However, contradicting our results, a recent Saudi study (2023) found higher acceptance among lower-income women (< 5,000 SAR), though this may reflect limited representation of higher-income participants (30). While higher-income individuals can more readily access procedures, interest spans across income levels, driven by desires to enhance self-confidence, appear younger, or increase attractiveness.

Prior research has linked social media use to greater interest in cosmetic procedures (1). Our study found that acceptance of cosmetic surgery was associated with an increase of 0.38 points for every additional hour of daily social media use (*p* = 0.035), with participants averaging 6.47 h daily. These findings align with research from Riyadh (2019) showing prolonged social media use increased cosmetic surgery interest among 53.2% of females (6). Similarly, studies in Rotterdam and Amsterdam (2022) found positive relationships between active social media usage and cosmetic procedure acceptance (1, 10, 31). However, because our data are cross-sectional, the reverse direction is equally plausible, whereby women already more accepting of cosmetic surgery preferentially seek out such content.

Our study found Snapchat and TikTok were the most-used platforms (35.2% and 38.2%, respectively), with ACSS scores higher by 4.55 points for Snapchat users (*p* = 0.026) and 4.34 points for TikTok users (*p* = 0.024) compared to Instagram users. These findings align with Arab et al.'s (6) Riyadh study, which identified Snapchat as the platform most strongly associated with cosmetic procedure decisions among Saudi female students. However, contradictory results were observed at King Abdulaziz Medical City's facial plastic clinic, where Instagram was most influential (55%), followed by Snapchat (40%) (11).

TikTok's algorithm delivers personalized beauty content to users who average 90 min daily on the app (32), while Snapchat's beauty filters create artificially enhanced appearances that users may seek to replicate through procedures (33). Both platforms showcase “before and after” transformations and feature influencers discussing their cosmetic enhancements, which may normalize these procedures and contribute to social pressure to achieve similar results (10). Research shows viewing attractive images on social media is associated with poorer body image (34) and greater cosmetic surgery interest (10).

Our study found following cosmetic specialists was associated with higher ACSS scores by 11.85 points (*p* < 0.001). This aligns with a 2019 Riyadh study where 66% of participants following plastic surgeons strongly considered procedures (6). Studies across Saudi Arabia confirm specialists' content, including before/after photos, influences cosmetic decisions (1, 7, 35). Additionally, our findings show women following beauty influencers have significantly higher ACSS scores (7.99 points, *p* < 0.001). This aligns with a 2022 Netherlands study showing positive correlation between following influencers who discuss cosmetic procedures and intention to undergo them, while following influencers without procedures correlated negatively (36). A Saudi Arabian study found 85.8% of males and 92.6% of females believe cosmetic procedures are popular among influencers, with many considering procedures to increase Snapchat followers (7).

Our analysis showed that women who posted selfies had significantly higher acceptance of cosmetic surgery, scoring 3.70 points more than non-posters (*p* = 0.012). Similarly, women using photo-editing applications showed significantly higher ACSS scores by 5.33 points (*p* < 0.001). These findings align with a 2019 Riyadh study where 54.1% of participants reported their desire to look better in selfies contributed to increasing cosmetic surgery trends (12). Similarly, a 2019 U.S. study found participants who edited facial features had higher ACSS consideration scores (by 1.74 points) than non-users, and those removing unedited photos had higher scores (by 0.62 points) (37). A 2022 Netherlands study confirmed frequency of Instagram filter use positively associated with greater acceptance of cosmetic procedures (36). However, a 2024 nationwide Saudi study found no significant association between selfies taken daily and desire for cosmetic surgery (*p* = 0.520), though desire to appear more attractive in selfies strongly influenced participants, making them nearly three times more likely to consider procedures (23). The association between photo editing and cosmetic surgery acceptance may reflect social pressure, whereby altered images are perceived as beauty standards, together with appearance dissatisfaction. As these data are cross-sectional, we cannot determine whether photo-editing behaviors foster acceptance or whether more accepting individuals are simply more inclined to edit their images.

Our findings indicate that women who compare their appearance to beauty influencers had significantly higher acceptance of cosmetic surgery (β = 3.81, *p* = 0.017). This aligns with a 2021 Italian study in which young women who compared their appearance to celebrity images on Instagram showed greater acceptance of cosmetic surgery (38). Social media frequently promotes idealized beauty standards, which may be associated with greater consideration of cosmetic surgery.

Furthermore, beauty influencers showcase the latest trends, helping to normalize these practices and making them appear more desirable. Additionally, a stronger desire to resemble influencers was associated with greater interest in cosmetic procedures (β = 7.97, *p* < 0.001). Moreover, a study conducted in Riyadh in 2019 with 816 participants found that 21.5% believed their happiness would increase if they looked more like influencers, while 18% expressed a serious willingness to undergo cosmetic procedures. These findings emphasize the influence of social media figures on individuals' self-esteem and perceptions of attractiveness, reflecting associations between digital exposure and beauty standards and attitudes toward cosmetic enhancements (6). Exposure to idealized influencer images may create unrealistic beauty standards, which could contribute to dissatisfaction with one's appearance and a greater interest in cosmetic enhancements. In addition, lower self-esteem and body-image concerns may be associated with greater acceptance of cosmetic procedure (17, 37). Consistent with our findings, a recent cross-sectional study of young social media users reported that higher popularity perceptions and greater social appearance anxiety were positively associated with more favorable attitudes toward aesthetic procedures, and that filter and image-manipulation use were significant correlates (39). This convergence reinforces the role of appearance-related anxiety and social comparison in shaping cosmetic surgery attitudes, while similarly being limited by a cross-sectional design.

Our findings may be relevant to several public health priorities discussed in the literature, such as promoting realistic beauty standards, media-literacy education, ethical cosmetic advertising, access to psychological support for individuals with high appearance-focused engagement, and school-based programs that strengthen self-esteem and resilience. As this was a cross-sectional study, these represent potential implications rather than evidence-based recommendations and would require confirmation in prospective or interventional research.

Future research should employ longitudinal and mixed-methods designs, multi-region sampling, and mediation or path-analysis models to clarify the temporal direction and underlying mechanisms of these associations, and to evaluate educational interventions. Because reverse causation is plausible; women already accepting of cosmetic surgery may preferentially seek beauty-related content; and unmeasured psychosocial factors may confound the findings, these results should be considered hypothesis-generating pending prospective studies.

### 4.1 Limitations

This study had several limitations. First, its cross-sectional design precludes causal inference and cannot establish the direction of the observed associations. Second, the convenience sampling method, conducted via social media apps, may have introduced selection bias by overrepresenting active online users and underrepresenting less connected women. Data were collected anonymously through publicly shared social media links, preventing verification of participant identity or unique responses; despite limiting one submission per device, duplicates cannot be ruled out. Anonymity also precluded calculating a response rate and may have reduced sample representativeness. Additionally, reliance on self-reported data may have introduced recall and social desirability biases. The use of a single measurement scale (ACSS) without complementary qualitative methods limited the depth of understanding regarding participants' motivations. Furthermore, the study did not control for potential confounding variables such as pre-existing body dysmorphic disorders or previous cosmetic procedures. This study did not include several psychosocial and experiential variables such as previous cosmetic procedures, prior consultations, body satisfaction, self-esteem, body dysmorphic symptoms, and exposure to cosmetic surgery advertising; that are known to affect cosmetic surgery acceptance. Future studies should include these factors to provide a more comprehensive understanding of the determinants of cosmetic surgery acceptance. Finally, as data collection was confined to the Jazan region, findings cannot be generalized to the broader Saudi Arabian population.

## 5 Conclusions

This study reveals significant associations between social media usage, demographic factors, and cosmetic surgery acceptance among women in the Jazan region of Saudi Arabia. The findings indicate that women with increasing age, higher income, and greater social media engagement show elevated acceptance of cosmetic surgery. Particularly relevant activities include following beauty experts, posting selfies, comparing oneself to influencers, and using photo-editing applications. These results suggest that digital platforms are associated with beauty standards and may relate to attitudes toward healthcare decisions in contemporary Saudi society. The implications may extend beyond individual choices to public health, pointing to areas; such as media literacy education, ethical guidelines for online content, and pre-procedure psychological assessment; that could be explored in future research and practice. Understanding these dynamics may help stakeholders encourage healthier body image and informed decision-making.This study highlights the association between social media engagement and women's acceptance of cosmetic surgery in Saudi Arabia. These insights may inform healthcare professionals and policymakers considering approaches to promote informed decisions, ethical counseling, and media literacy. For providers, they underscore the value of ethical patient counseling so that decisions regarding cosmetic procedures reflect personal choice. Future research is recommended to explore potential causal relationships through longitudinal designs and randomized controlled trials, and to evaluate interventions that support healthier body image.

## Data Availability

The data presented in this study are available on request from the corresponding author.
